# Laryngitis Caused by *Mycobacterium abscessus* Subspecies *massiliense* Infection

**DOI:** 10.31662/jmaj.2023-0202

**Published:** 2024-06-10

**Authors:** Yu Kurahara, Yasuaki Shimatani

**Affiliations:** 1 Clinical Research Center, NHO Kinki Chuo Chest Medical Center, Osaka, Japan; 2 Department of Infectious Disease, NHO Kinki Chuo Chest Medical Center, Osaka, Japan; 3 Department of Clinical Laboratory, NHO Kinki Chuo Chest Medical Center, Osaka, Japan

**Keywords:** *Mycobacterium abscessus*, *Mycobacterium massiliense*, laryngitis

A 23-year-old woman visited our hospital with a 1-month history of laryngitis and presented with a sore throat 3 weeks after being diagnosed with COVID-19. Laryngoscopy revealed extensive erosion and a white coating in the larynx (Figure 1a). Rapidly growing mycobacteria were isolated from laryngeal tissue on 2% Ogawa egg medium (Figure 1b) and identified as *Mycobacterium abscessus* subspecies *massiliense* (Mma) using MALDI-TOF MS (Bruker Daltonics, Billerica, MA, USA) and DNA Chromatography MABC/*erm*(41) (Kaneka, Osaka, Japan). The patient’s skin did not show any signs of Mma infection, and no lung lesions were identified. She was later diagnosed with HIV infection. Her CD4 count was 376 cells/mm^3^.

**Figure 1.**
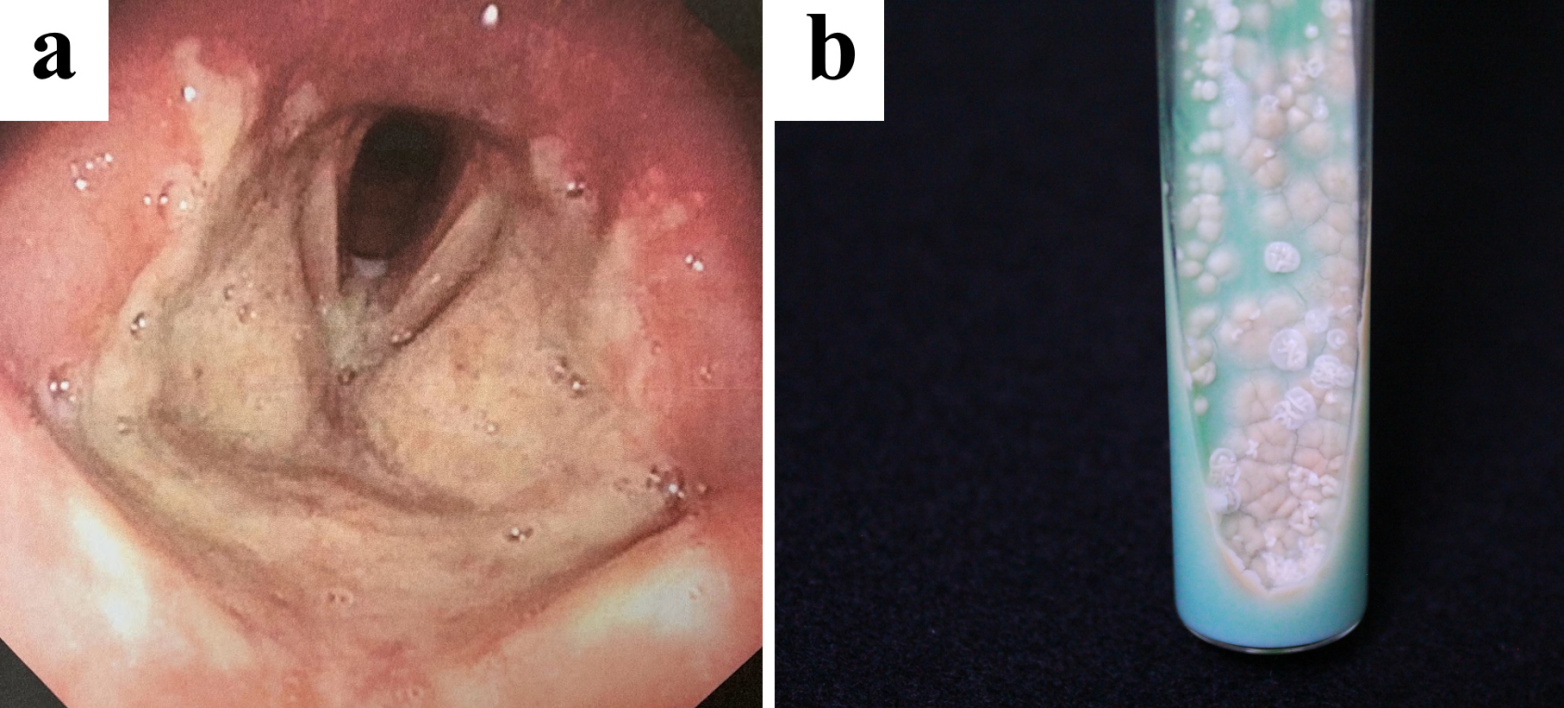
(a) Laryngoscopy revealing extensive erosion and a white coating in the larynx. (b) *Mycobacterium abscessus* subspecies *massiliense* cultured on 2% Ogawa egg medium.

This patient’s main symptom was initially suspected to be a sequela of COVID-19, but the eventual diagnosis was Mma laryngitis associated with HIV infection. Mma infections in the head and neck are rare ^(1)^. Clinicians should be aware that laryngitis that is resistant to therapy may in fact be a laryngeal nontuberculous mycobacterial infection, especially in immunocompromised patients. Furthermore, bacteriological investigation aimed at identifying specific pathogens is crucial for patients with atypical laryngitis.

## Article Information

### Conflicts of Interest

None

### Author Contributions

YK wrote the draft of the manuscript and contributed to patient care. YS contributed to bacteriological examinations.

### Informed Consent

Written informed consent was obtained from the patient by the corresponding author. The signed consent forms have been retained by the patient. Details of the patient have been anonymized as much as possible.

### Approval by Institutional Review Board (IRB)

This report did not require IRB approval.
